# Anti-infective Effects of a Fish-Derived Antimicrobial Peptide Against Drug-Resistant Bacteria and Its Synergistic Effects With Antibiotic

**DOI:** 10.3389/fmicb.2020.602412

**Published:** 2020-11-23

**Authors:** Yue Chen, Jing Wu, Honglan Cheng, Yue Dai, Yipeng Wang, Hailong Yang, Fei Xiong, Wei Xu, Lin Wei

**Affiliations:** ^1^ Jiangsu Key Laboratory of Infection and Immunity, Institutes of Biology and Medical Sciences, Soochow University, Suzhou, China; ^2^ School of Basic Medical Sciences, Kunming Medical University, Kunming, China; ^3^ The Second Affiliated Hospital of Soochow University, Suzhou, China; ^4^ Department of Biopharmaceuticals, College of Pharmaceutical Sciences, Soochow University, Suzhou, China

**Keywords:** Erythroculter ilishaeformis, antimicrobial peptide, liver-expressed antimicrobial peptide 2, aquatic bacteria, synergistic effect, drug resistance

## Abstract

Antimicrobial peptides (AMPs) play pivotal roles in protecting against microbial infection in fish. However, AMPs from topmouth culter (*Erythroculter ilishaeformis*) are rarely known. In our study, we isolated an AMP from the head kidney of topmouth culter, which belonged to liver-expressed antimicrobial peptide 2 (LEAP-2) family. Topmouth culter LEAP-2 showed inhibitory effects on aquatic bacterial growth, including antibiotic-resistant bacteria, with minimal inhibitory concentration values ranging from 18.75 to 150 μg/ml. It was lethal for *Aeromonas hydrophila* (resistant to ampicillin), and took less than 60 min to kill *A. hydrophila* at a concentration of 5 × MIC. Scanning electron microscope (SEM) and SYTOX Green uptake assay indicated that it impaired the integrity of bacterial membrane by eliciting pore formation, thereby increasing the permeabilization of bacterial membrane. In addition, it showed none inducible drug resistance to aquatic bacteria. Interestingly, it efficiently delayed ampicillin-induced drug resistance in *Vibrio parahaemolyticus* (sensitive to ampicillin) and sensitized ampicillin-resistant bacteria to ampicillin. The chequerboard assay indicated that topmouth culter LEAP-2 generated synergistic effects with ampicillin, indicating the combinational usage potential of topmouth culter LEAP-2 with antibiotics. As expected, topmouth culter LEAP-2 significantly alleviated ampicillin-resistant *A. hydrophila* infection *in vivo*, and enhanced the therapeutic efficacy of ampicillin against *A. hydrophila in vivo*. Our findings provide a fish innate immune system-derived peptide candidate for the substitute of antibiotics and highlight its potential for application in antibiotic-resistant bacterial infection in aquaculture industry.

## Introduction

To improve the dietary protein ingestion for the increasing world population, the world aquaculture is experiencing a rapid expansion, and fish farming comprises an important source of protein demands for people (Yousefi et al., 2018). In order to achieve a better profitability, fish culture systems have been changed into more complicated systems with higher density that corresponds to a higher production level (Yousefi et al., 2018). However, the intensive culture of fish simultaneously brings more and more fish diseases, which cause severe economic losses (Xiong et al., 2019). Especially, bacterial infection comprises one of the major fish diseases (Chen et al., 2019). Up to now, the usage of antibiotics is still the most favorable strategy to control bacterial infection in fish (Chen et al., 2019). The wide usage of antibiotics has resulted in the increasing spread of antibiotic-resistant bacteria in farmed fish and antibiotic residues in farmed fish food. Considering the strict regulations for administration of antibiotic for fish disease treatment, we are obligated to develop novel strategies to control the emergence of antibiotic-resistant bacteria in farmed fish and antibiotic residues in farmed fish food. Therefore, many researchers have explored the potential application of various types of substitutes for antibiotics over the past decades. Among them, antimicrobial peptides (AMPs) gained more and more research interests because of their broad antimicrobial activities, effective immunomodulatory activities, and none induced drug resistance risk.

AMPs, also known as host defense peptides (HDPs), are pivotal molecules of innate immunity that provide the first line of host defense against microbial invasion (Zasloff, 2002, 2019). AMPs are widely expressed in vertebrates, including fishes, amphibians, reptiles, birds and mammals (Wei et al., 2013). As a lower vertebrate, the innate immune system is a fundamental defense mechanism of fish (Shabir et al., 2018), and it serves as a bridge between innate and adaptive immunity, which is considered as an excellent model for innate immunity and comparative immunology investigations (Liang et al., 2013; Zhu et al., 2013). So far, several families of AMPs have been characterized from fishes, including piscidins, cathelicidins, defensins, and liver-expressed antimicrobial peptide 2 (LEAP-2, also known as hepcidins; Shabir et al., 2018). Among these AMPs, piscidins, and cathelicidins are linear peptides, while defensins and LEAP-2 contain intramolecular disulfide bonds. They were found to disrupt pathogenic bacterial membranes (Chen et al., 2019). In addition, they were also observed to regulate innate immune response (Chen et al., 2019). These properties of AMPs effectively reduce the possibility of development of resistance against bacteria.

LEAP-2 are a cysteine-rich cationic peptide family and a highly conserved peptide family in different species (Yang et al., 2014). They share four cysteine residues at conserved positions and even share several conserved amino acid sequences (Yang et al., 2014). The four conserved cysteine residues usually formed two intramolecular disulfide bonds linked in Cys1-Cys3 and Cys-2-Cys4 patterns (Chen et al., 2019). So far, a series of LEAP-2 have been characterized from several fish species, including channel catfish (*Ictalurus punctatus*; Bao et al., 2006), grass carp (*Ctenopharyngodon idella*; Liu et al., 2010), common carp (*Cyprinus carpio L*.; Yang et al., 2014), blunt snout bream (*Megalobrama amblycephala*; Liang et al., 2013), rainbow trout (*Oncorhynchus mykiss*; Zhang et al., 2004), miiuy croaker (*Miichthys miiuy*; Liu et al., 2014), teleost fish (*Plecoglossus altivelis*; Li et al., 2015), mudskipper (*Boleophthalmus pectinirostris*; Chen et al., 2016), and barbel steed (*Hemibarbus labeo*; Chen et al., 2019). LEAP-2 play critical roles in host innate immune response against pathogenic invasion in these fish species (Yang et al., 2014). They were demonstrated to have direct antimicrobial activities against Gram-positive bacteria, Gram-negative bacteria and fungi (Yang et al., 2014). However, little is known about its anti-infective effects and its interactive effects with antibiotics against antibiotic-resistant bacterial infection.

Topmouth culter (*Erythroculter ilishaeformis*) is a kind of freshwater fish belonging to the genera of Culter fishes (Dong et al., 2017). It is one of the important economical fishes in Taihu Lake, which is the third largest freshwater lake in China (Dong et al., 2018). Topmouth culter, whitebait (*Neosalanx taihuensis*), and white shrimp (*Fenneropenaeus chinensis*) are well-known as “three whites” of Taihu Lake that provide a lot of dietary protein for people (Dong et al., 2017). Due to overfishing, the wild topmouth culter is almost exhausted. Recently, topmouth culter exhibits great cultural potential with rapid growth rate. However, research on the innate immunity of topmouth culter is rarely conducted. In this study, we isolated and cloned an AMP (LEAP-2) from topmouth culter. The effects of topmouth culter LEAP-2 on aquatic pathogenic bacteria were tested by minimal inhibitory concentration (MIC) assay, bacterial killing kinetics assay, minimal inhibitory concentration (SEM) observation, and SYTOX Green uptake assay. The possibility of inducible drug resistance of topmouth culter LEAP-2 against aquatic pathogenic bacteria was evaluated. The combinational usage of topmouth culter LEAP-2 and traditional antibiotic was investigated both *in vitro* and *in vivo*. Our findings provide a topmouth culter-derived AMP for controlling antibiotic-resistant bacterial infection-induced diseases in cultured fishes.

## Materials and Methods

### Animals and Ethics Approval

Healthy topmouth culter (*E. ilishaeformis*; body weight 350–450 g) were purchased from a commercial farm in Suzhou, China. Topmouth culter were reared in circulating filtered water tanks at the temperature of at 20–25°C. All surgery of topmouth culter were performed after they were anesthetized in 0.2% tricaine and euthanized by incubating in ice water for 15 min. C57BL/6 mice (female, 18–20 g) were purchased from Shanghai Slac Animal Co. Inc. and housed in a pathogen-free facility. All surgery of mice were performed under sodium pentobarbital anesthesia with minimum fear, anxiety, and pain. Animal experiments were approved by the Animal Care and Use Committee and the Ethical Committee of Soochow University (SYXK2017-0043).

### Bacteria

Aquatic pathogenic bacterial strains, including *Aeromonas sobria*, *Aeromonas hydrophila*, *Vibrio harveyi*, *Vibrio parahaemolyticus*, *Vibrio anguillarum*, *Vibrio vulnificus*, *Vibrio splendidus*, and *Vibrio cholera*, were collected from our previous studies (Guo et al., 2017; Chen et al., 2019), and cultured in nutrient broth (Oxoid, UK) at 37°C.

### Peptide Purification

After healthy topmouth culter (*E. ilishaeformis*) were anesthetized in 0.2% tricaine and euthanized *via* incubation in ice water for 15 min, head kidneys were collected and homogenized in 0.1 M phosphate buffer (PBS; pH 6.0, 1 g tissue/ml) containing 1% (v/v) protease inhibitor cocktail (Sigma-Aldrich). The homogenates were centrifuged at 12,000 × *g* for 10 min, and the supernatants were harvested and lyophilized. Lyophilized samples were dissolved in 9 ml phosphate buffer (0.1 M, pH 6.0, OD280 = 56.3). The dissolved samples were subjected to a Sephadex G-50 (Superfine, Amersham Biosciences, 2.6 cm × 100 cm) gel filtration column. phosphate buffer (0.1 M, pH 6.0) was used as eluted buffer at a flow rate of 3.0 ml/10 min. The eluted fractions were measured at 280 nm, and the fractions with antimicrobial activity were pooled. The fractions with antimicrobial activity were then subjected to a C18 reversed-phase high-performance liquid chromatography column (RP-HPLC, 5 μm particle size, 110 Å pore size, 250 mm × 4.6 mm, Gemini, CA, United States) using a linear gradient of 0–60% acetonitrile supplemented with 0.1% (v/v) trifluoroacetic acid/water. The eluted peaks were collected for antimicrobial assay, and the eluted peak with antimicrobial activity was spotted onto a matrix-assisted laser desorption ionization time-of-flight (MALDI-TOF) plate for purity assay. The purified peptide was applied to a protein sequencer (PPSQ-31A; Shimadzu, Kyoto, Japan) following manufacturer’s instruction.

### cDNA Cloning

Total RNA from the head kidney of topmouth culter was extracted using Trizol reagent (Life Tech, United States). Head kidney cDNA library was constructed using a SMART™ PCR cDNA synthesis kit (Clontech, CA). Two primers, a sense primer (5' PCR primer, 5'-AAGCAGTGGTATCAACGCAGAGT-3', provided by the cDNA library construction kit) and an antisense primer (S1(5'-A(A/G)CAT(G/A/T)ATIC(G/T)CCA(A/G/C/T)A(A/G)(A/G/C/T)GG-3'), designed from the amino acid sequence of topmouth culter LEAP-2 obtained by Edman degradation), were used to screen the 5' fragment of cDNA encoding topmouth culter LEAP-2. Then, a sense primer (5'-ATGCAGACCCACCCCAACAG-3', designed according to the 5' fragment of cDNA) and an antisense primer (3' PCR primer, 5'-ATTCTAGAGGCCGAGGCGGCCGACATG-3', provided by the cDNA library construction kit) were used to screen the full-length cDNA that encodes topmouth culter LEAP-2. The PCR conditions were 2 min at 95°C, and 28 cycles of 10 s at 92°C, 30 s at 56°C, and 30 s at 72°C, and followed by 10 min extension at 72°C. The PCR products were cloned into pGEM-T Easy vector (Promega, Madison, WI, United states), and positive clones were selected for DNA sequencing performed by Genewiz Co. Ltd. (Suzhou, China).

### Sequence Analysis

The deduced amino acid sequence of topmouth culter LEAP-2 precursor was translated from the cDNA sequence using the ExPASy Translate Tool.^1^ Blast search was performed with Blastx provided by NCBI.^2^ Multiple alignment of amino acid sequence of topmouth culter LEAP-2 with its homologues was performed with ClustalW.^3^ Theoretical isoelectric point (pI) and molecular weight (MW) were calculated by an online tool^4^ (Wei et al., 2015).

### Antimicrobial Assay

A standard 2-fold broth microdilution method was used to evaluate the antimicrobial activity of topmouth culter LEAP-2 against aquatic pathogenic bacteria. Aquatic pathogenic bacteria were diluted with fresh nutrient broth (Oxoid, UK) to 10^5^ CFU/ml after bacteria were cultured to exponential phase in nutrient broth. A series of topmouth culter LEAP-2 dilutions were prepared in 96-well plates (50 μl/well), and an equal volume of bacterial dilution was added. The plates were cultured at 37°C for 18 h. Ampicillin was used as positive control in antimicrobial assay. The MIC at which no visible bacterial growth occurred was recorded as MIC value (Wei et al., 2013).

### Bacterial Killing Kinetics Assay

Bacterial killing kinetic assay was tested according to the method described previously (Chen et al., 2019). Briefly, *A. hydrophila* (an ampicillin resistant strain, MIC > 200 μg/ml) in exponential phase were diluted using nutrient broth at density of 10^5^ CFU/ml. Topmouth culter LEAP-2 (5 × MIC, 93.75 μg/ml), ampicillin (1 mg/ml) or an equal volume of phosphate-buffered saline (PBS, vehicle) was added to bacterial dilution and incubated at 37°C for 0, 10, 20, 30, 45, 60, 90, 120, and 180 min, respectively. At each time point, 50 μl of bacterial solution was diluted 1,000 times, and 50 μl of bacterial dilution was coated on nutrient broth agar plates. Bacterial colonies were recorded after culture at 37°C for 12 h.

### Scanning Electron Microscope Assay

To examine if topmouth culter LEAP-2 impaired the bacterial surface morphology, *A. hydrophila* were cultured in nutrient broth to exponential phase, washed with PBS, and suspended in PBS (about 5 × 10^6^ CFU/ml). Topmouth culter LEAP-2 (5 × MIC) was added into the bacterial suspension and incubated at 37°C for 30 min. After centrifuged at 1,000 × g for 10 min, *A. hydrophila* were fixed for SEM assay following standard operating conditions. The surface morphology was observed with a Hitachi SU8010 SEM (Japan) following the manufacturer’s instruction.

### SYTOX Green Assay

SYTOX Green assay was performed to investigate the membrane integrity of *A. hydrophila* after incubation with topmouth culter LEAP-2 as described previously (Jin et al., 2016). Briefly, the ampicillin-resistant *A. hydrophila* were suspended in PBS (10 mM, pH 7.4) at a density of 10^6^ CFU/ml, and incubated with topmouth culter LEAP-2 (1 × MIC), ampicillin (1 mg/ml), or PBS (vehicle) in the presence of SYTOX Green nucleic acid fluorescent dye (0.1 μM, Thermo fisher scientific, United States) at 37°C for 15 min on a shaking table. Fluorescence intensity was monitored by a FlexStation microplate reader per 2 min using an excitation of 488 nm and emission of 530 nm. Results were presented as relative fluorescence units (RFU).

### Drug Resistance Test

*Vibrio parahaemolyticus* were sensitive to ampicillin, and were selected for drug resistance test against topmouth culter LEAP-2 and ampicillin. Briefly, drug resistance was induced in *V. parahaemolyticus* by repeated treatment with ampicillin or topmouth culter LEAP-2 for 10 passages and tested *via* MIC measurement. The same volume of vehicle (PBS) was used as control. For each passage, *V. parahaemolyticus* were exposed to a sub-MIC concentration of ampicillin or topmouth culter LEAP-2 (1/8 MIC at that particular passage) until growing to the log phase, and the MIC values of topmouth culter LEAP-2 and ampicillin against *V. parahaemolyticus* were determined as mentioned above.

### Combinational Usage of Topmouth Culter LEAP-2 With Ampicillin

To explore the potential of the combinational usage of topmouth culter LEAP-2 with antibiotic, we tested the ampicillin-induced drug resistance in *V. parahaemolyticus* as mentioned above in the presence of topmouth culter LEAP-2. Briefly, *V. parahaemolyticus* were passaged for 10 times in the presence of ampicillin and topmouth culter LEAP-2. For each passage, *V. parahaemolyticus* was exposed to a sub-MIC concentration of ampicillin (1/8 MIC at that particular passage) and a constant concentration of topmouth culter LEAP-2 (18.75 μg/ml, 1/4 MIC of LEAP-2 against *V. parahaemolyticus*) until growing to the log phase, and the MIC values of ampicillin against *V. parahaemolyticus* were determined *via* MIC assay.

In addition, we also tested the MIC values of ampicillin against aquatic pathogenic bacteria (including ampicillin-sensitive and ampicillin-resistant strains) in the presence of topmouth culter LEAP-2 at a concentration of 1/4 MIC of LEAP-2 against respective bacterial strain.

### Chequerboard Assay

Interactive interactions between LEAP-2 and ampicillin were tested by a chequerboard assay according to the method described previously (Yang et al., 2019). In brief, a series of 2-fold LEAP-2 or ampicillin dilutions were prepared, and equal volume of LEAP-2 (50 μl/well) and LEAP-2 (50 μl/well) were mixed in a well of 96-well plate. Then bacteria (10^5^ CFU/ml, 100 μl/well) was added and cultured at 37°C for 18 h bacterial growth was recorded by a microplate reader at 600 nm.

### *In vivo* Antimicrobial Assay

The anti-infective effect against antibiotic-resistant bacteria of topmouth culter LEAP-2 in fish was tested according to method described previously with slight modification (Chen et al., 2019). Topmouth culter were randomly divided into four groups, and were intraperitoneally challenged with *A. hydrophila* (100 μl, 2 × 10^7^ CFU/ml). After bacterial challenge, topmouth culter in each group were immediately intraperitoneally administrated with LEAP-2 (10 mg/kg), ampicillin (10 mg/kg), LEAP-2 and ampicillin used in combination (10 mg/kg each), or an equal volume of PBS (vehicle) respectively. At 6 h post infection, peritoneal lavage was collected by intraperitoneal injection of 5 ml PBS. Its anti-infective effect against antibiotic-resistant bacteria was also evaluated in mice as described previously (Wei et al., 2018). C57BL/6 mice (female, 18–20 g, *n* = 6) were intraperitoneally challenged with *A. hydrophila* (resistant to ampicillin, 2 × 10^7^ CFUs/mouse). Topmouth culter LEAP-2 (10 mg/kg) or an equal volume of PBS (vehicle) was intraperitoneally administered into mice post bacterial infection. At 18 h post infection, peritoneal lavage was collected by intraperitoneal injection of 2 ml PBS. A series of 10-fold dilutions of fish and mouse peritoneal lavage were prepared, and 50 μl of different dilutions was plated on nutrient broth agar plates. The CFUs were counted after the agar plates were cultured at 37°C for 18 h.

### Synthetic Peptides

Synthetic peptide was purchased from Synpeptide Co. Ltd. (Shanghai, China). The purity of the synthetic peptide was analyzed by RP-HPLC and MALDI-TOF MS.

### Statistical Analysis

Statistical analysis was performed using Student’s t-tests or one-way ANOVA provided by GraphPad Prism software (GraphPad Software Inc., La Jolla, CA, United States). Data were presented as mean ± standard deviation from three independent experiments. *p* < 0.05 was considered as statistically significant of difference between the groups.

## Results

### A Novel AMP, LEAP-2, Was Isolated From the Head Kidney of Topmouth Culter

**Figure 1:**
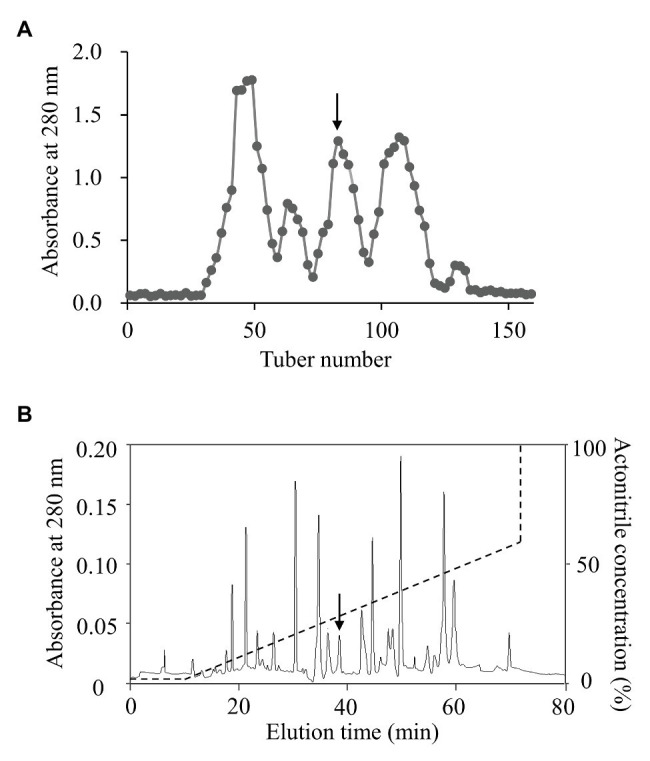
Purification of AMP from topmouth culter. **(A)** Sephadex G-50 gel filtration. The homogenates of head kidney were applied to Sephadex G-50 gel filtration column and eluted with 0.1 M PBS at a flow rate of 3 ml/10 min, and the fraction containing antimicrobial activity was marked by an arrow. **(B)** The antimicrobial fraction from Sephadex G-50 gel filtration was separated by a C18 RP-HPLC column with the indicated gradient of acetonitrile in 0.1% (v/v) trifluoroacetic acid in water, and the eluted peak containing antimicrobial activity was marked by an arrow.

To isolate topmouth culter (*E. ilishaeformis*)-derived AMPs, head kidney, an important immune organ of topmouth culter, was collected and homogenized. The condensed supernatants of head kidney homogenates were first separated by Sephadex G-50 gel filtration. As shown in Figure 1A, the condensed supernatants were separated into five fractions, and the fraction containing antimicrobial activity was indicated by an arrow. The antimicrobial fraction from Sephadex G-50 gel filtration was then subjected to a C18 RP-HPLC column (Figure 1B). The eluted peak containing antimicrobial activity was marked with an arrow. The purified AMP was subjected to mass spectrometry analysis and had an observed MW of 4652.17 Da (Table 1). The N-terminal amino acid sequence of the purified peptide was determined as MTPLWRIMLLFKPHALCQNNY by Edman degradation.

**Table 1:** Physico-chemical parameters of topmouth culter LEAP-2.

Amino acids	Net charge	Intramolecular disulfide bond	pI*^a^*	Theoretical MW*^b^*	Observed MW
41	+3	2	8.91	4652.56	4652.17

a Isoelectric point.

b Molecular weight (Da).

### The Purified AMP From Topmouth Culter Belongs to LEAP-2 Family

**Figure 2:**
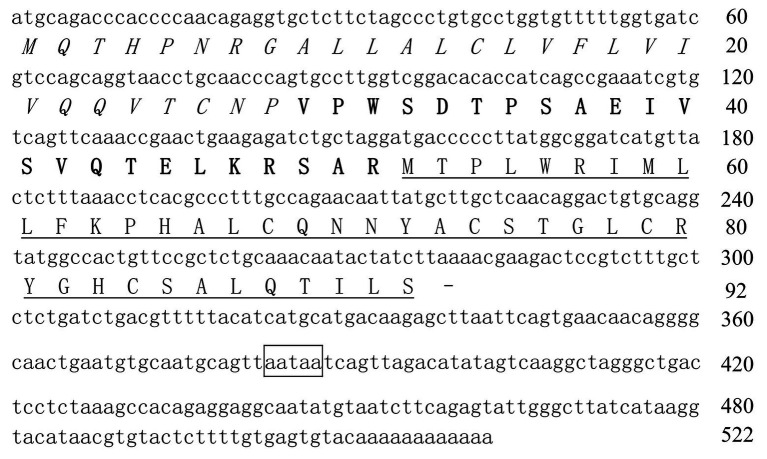
The nucleotide sequence encoding topmouth culter LEAP-2 precursor. The amino acids of signal peptide are italic, the amino acids of prodomain are bold, and the amino acids of mature peptide are underlined. Line segment (−) indicates stop codon. The putative polyadenylation site (aataa) is boxed.

**Figure 3:**
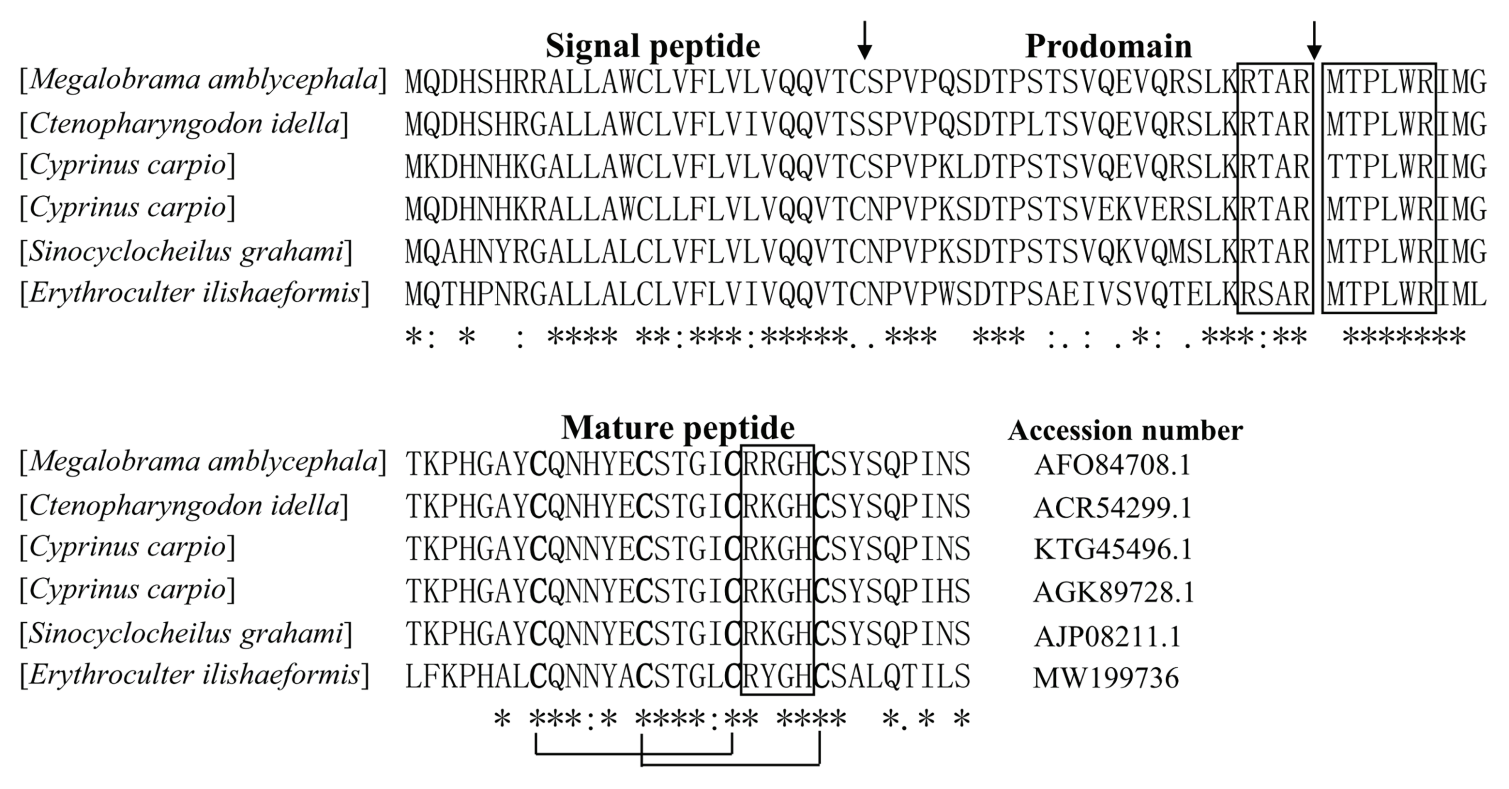
Multiple alignment of the amino acid sequences of topmouth culter LEAP-2 and its homologues. The identical sites (^*^), conserved sites (:), and less conserved sites (.) were indicated in the multiple alignment of topmouth culter LEAP-2 with its homologues, the accession numbers of the selected homologues were listed following each sequence. The predicted cleavage site for the signal peptide and the cleavage site for mature peptide were marked with an arrow. The boxes indicated “RXXR,” “MTPLWR,” and “RXGH” motifs respectively. The four conserved cysteine residues in the mature peptide are bold. Two cysteine residues joined by a solid line represented a disulfide bond.

According to N-terminal amino acid sequence of the purified peptide by Edman degradation, we designed primer and tried to clone the cDNA sequence from the head kidney of topmouth culter, and we successfully obtained the open reading frame of the purified AMP (Figure 2). The cDNA sequence that encoded the precursor of the purified AMP contained 522 base pairs (Figure 2; GenBank accession number, MW199736). The precursor of topmouth culter LEAP-2 was composed of 92 amino acid residues. Blast search indicated that the precursor is a member of LEAP-2 AMP family that contained a signal peptide (italic), a prodomain (bold), and a mature peptide (underlined; Figure 2). The deduced mature peptide of topmouth culter LEAP-2 is composed of 41 amino acids (Figure 2, Table 1), and the N-terminal amino acid sequence of the deduced mature LEAP-2 is consistent with that (MTPLWRIMLLFKPHALCQNNY) obtained by Edman degradation. The mature peptide of topmouth culter LEAP-2 has a theoretical MW of 4652.56 Da, net charges of +3, and a theoretical isoelectric point of 8.91 (Table 1). The theoretical MW of mature LEAP-2 matched well with the observed MW. The alignment of topmouth culter LEAP-2 precursor with selected cyprinid fish LEAP-2 precursors indicated that there are a conserved “RXXR” amino acid sequence before the cleavage site separating the prodomain and the mature LEAP-2 peptide, a conserved “MTPLWR” amino acid sequence at the N-terminal of mature LEAP-2 peptide, and a conserved “RXGH” amino acid sequence at the C-terminal of mature LEAP-2 peptide as described previously (Figure 3; Chen et al., 2019). In addition, the mature LEAP-2 peptide of topmouth culter contains four conserved cysteine residues that form two intramolecular disulfide bonds, and the four cysteine residues are probably linked in Cys1-Cys2 and Cys2-Cys4 patterns as described previously (Figure 3; Chen et al., 2019).

### Topmouth Culter LEAP-2 Showed Antimicrobial Activity Against Aquatic Pathogenic Bacteria

To investigate the antimicrobial effects of LEAP-2, we detected the MIC values of topmouth culter LEAP-2 against aquatic pathogenic bacteria. As shown in Table 2, all the tested aquatic bacterial strains were sensitive to topmouth culter LEAP-2, and the MIC values ranged from 18.75 to 150 μg/ml. Among these tested aquatic bacterial strains, *A. sobria*, *A. hydrophila*, *V. anguillarum*, *V. vulnificus*, *V. splendidus*, and *V. cholerae* were resistant to ampicillin with MIC values higher than 200 μg/ml, but these ampicillin-resistant aquatic bacterial strains were all sensitive to topmouth culter LEAP-2 with MIC values ranging from 18.75 to 150 μg/ml. These results revealed that topmouth culter LEAP-2 showed antimicrobial activity against aquatic pathogenic bacteria, including antibiotic-resistant bacterial strains.

**Table 2:** Antimicrobial activities of topmouth culter LEAP-2 *in vitro*.

Microorganisms	MIC (μg/ml)*^a^*	
	LEAP-2	Ampicillin
*Aeromonas sobria*	75 (16.1 μM)	>200
*Aeromonas hydrophila*	18.75 (4.0 μM)	>200
*Vibrio harveyi*	75 (16.1 μM)	37.5
*Vibrio parahaemolyticus*	75 (16.1 μM)	18.75
*Vibrio anguillarum*	150 (32.2 μM)	>200
*Vibrio vulnificus*	37.5 (8.1 μM)	>200
*Vibrio splendidus*	150 (32.2 μM)	>200
*Vibrio cholerae*	37.5 (8.1 μM)	>200

These concentrations represent mean values from three independent experiments. Topmouth culter LEAP-2 were dissolved in PBS (2 mg/ml), and a series of 2-fold peptide dilution were prepared with nutrient broth in 96-well plates (50 μl/well), and bacterial dilution (10^5^ CFU/ml, 50 μl/well) was added. Ampicillin was used as positive control. After the plates were cultured at 37°C for 18 h, the MIC values were recorded.

a MIC, minimal inhibitory concentration.

### Topmouth Culter LEAP-2 Had a Rapid Bactericidal Speed

To evaluate the antimicrobial efficacy of topmouth culter LEAP-2, its bacterial killing kinetics against *A. hydrophila* (an ampicillin resistant strain, MIC > 200 μg/ml) was evaluated. As listed in Table 3, topmouth culter LEAP-2 rapidly inhibited the bacterial growth as compared to PBS. Topmouth culter LEAP-2 (5 × MIC, 93.75 μg/ml, equal to 20.2 μM) took less than 60 min to kill all the bacteria, and it was proved to be lethal for *A. hydrophila* since *A. hydrophila* were not capable of resuming growth on agar plates. However, ampicillin (1 mg/ml, equal to 2862.0 μM) could not completely kill *A. hydrophila* until incubation for 180 min. On contrary, *A. hydrophila* dramatically growed after the addition of ampicillin, and the CFUs increased from 6.7 × 10^4^ to 9.6 × 10^5^ after the bacteria were incubated with ampicillin (1 mg/ml) for 180 min, which again indicated that this strain of *A. hydrophila* is resistant to ampicillin. These results suggested that topmouth culter LEAP-2 had a rapid bactericidal speed against antibiotic-resistant aquatic pathogenic bacteria.

**Table 3:** Killing kinetics of of topmouth culter LEAP-2 against *Aeromonas hydrophila*.

Amount of bacteria cocultured with different samples for different time (×10^3^, CFUs/ml)*^a^*									
Samples	Time (min)								
	0	10	20	30	45	60	90	120	180
LEAP-2	65 ± 5.3	59 ± 7.4	40 ± 8.4	28 ± 5.9	11 ± 3.5	0	0	0	0
Ampicillin	67 ± 7.5	69 ± 8.1	70 ± 7.2	72 ± 6.9	85 ± 6.9	123 ± 10.7	197 ± 12.6	378 ± 41.5	961 ± 87.3
PBS	62 ± 7.9	63 ± 9.2	77 ± 8.3	84 ± 11.6	149 ± 18.2	218 ± 30.1	324.3 ± 39.2	542 ± 75.4	1,527 ± 271.7

Topmouth culter LEAP-2 (5 × MIC, 93.75 μg/ml), ampicillin (1 mg/ml) or an equal volume of PBS (vehicle) was added to *Aeromonas hydrophila* (10^5^ CFU/ml) suspension in nutrient broth and incubated at 37°C. At each indicated time points, 50 μl of bacterial solution was diluted 1,000 times using nutrient broth, and 50 μl of bacterial dilution were coated on nutrient broth agar plates. After culture at 37°C for 12 h, the CFUs was counted.

a These CFUs represent mean values ± SD from three independent experiments.

### Topmouth Culter LEAP-2 Impaired the Bacterial Surface Morphology

**Figure 4:**
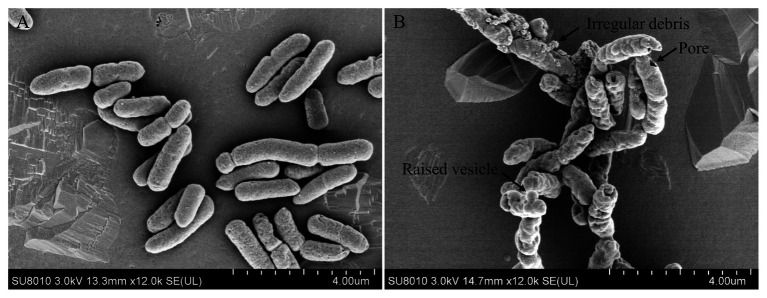
Effects of topmouth culter LEAP-2 on the surface morphology of *A. hydrophila. A. hydrophila* (about 5 × 10^6^ CFU/ml) were incubated with PBS (vehicle; A) or topmouth culterLEAP-2 (5 × MIC; B) at 37 °C for 30 min. The surface morphology was observed with a Hitachi SU8010 scanning electron microscope. The typical alterations that formed on bacterial surface, including raised vesicle, irregular debris, and pore, were marked with arrows as indicated in B.

Antimicrobial peptides were usually membrane-active agents, which lead to the alteration of bacterial membrane morphology (Khara et al., 2014). To see if topmouth culter LEAP-2 altered the membrane morphology of aquatic pathogenic bacteria, we observed the surface morphology of *A. hydrophila* by SEM after bacteria were exposed to PBS (vehicle, control) or topmouth culter LEAP-2. As shown in Figure 4, PBS-exposed *A. hydrophila* exhibited a regular, smooth, and intact surface (Figure 4A). Whereas LEAP-2-exposed *A. hydrophila* showed a significant change of surface morphology with raised vesicle and pore formation in the surface, and the bacterial surface was covered with irregular debris (Figure 4B). The results indicated that topmouth culter LEAP-2 exhibited direct antimicrobial activity by membrane-disruption mechanism.

### Topmouth Culter LEAP-2 Induced Bacterial Membrane Permeabilization

**Figure 5:**
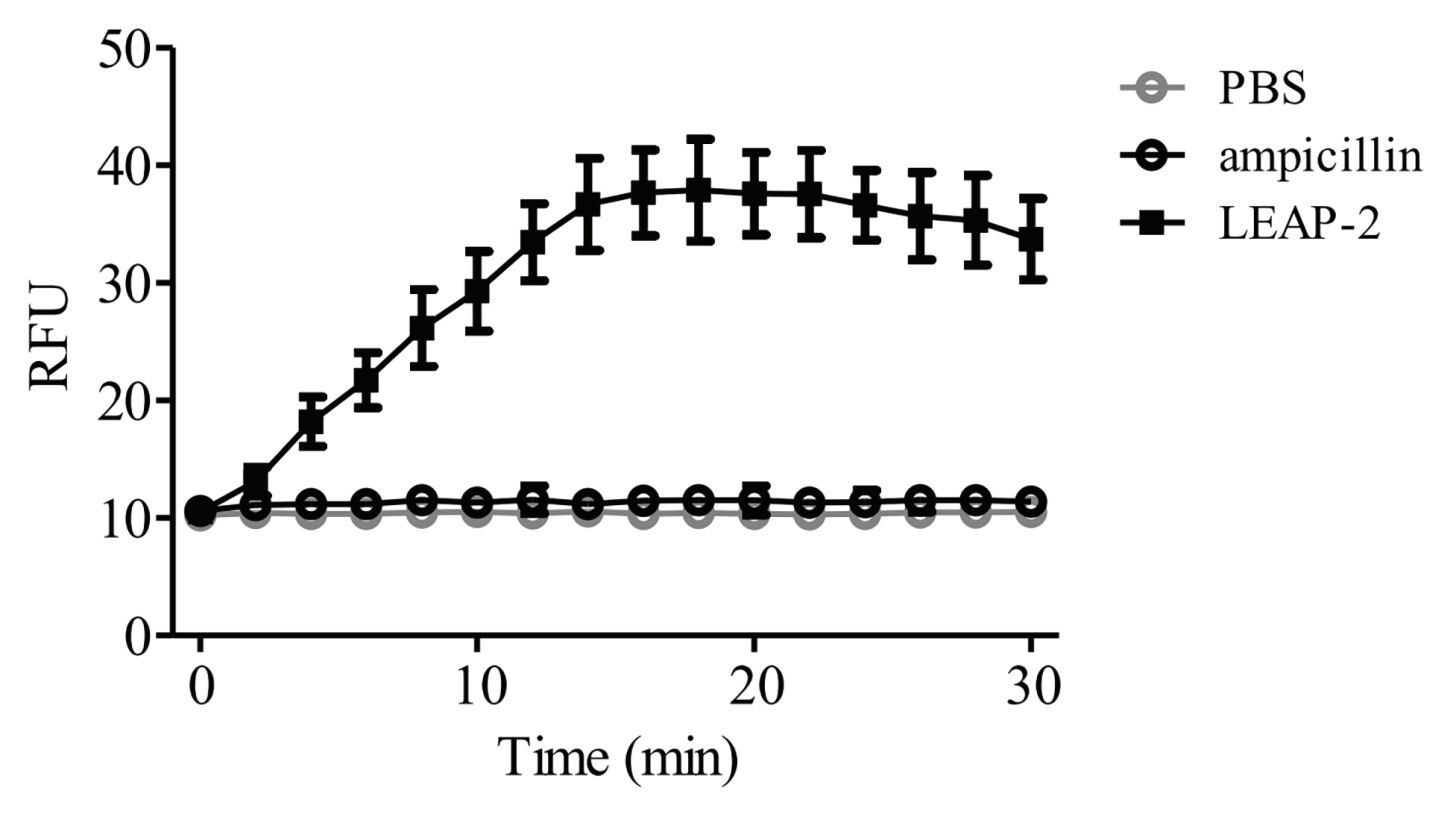
Effects of topmouth culter LEAP-2 on membrane permeabilization of *A. hydrophila*. *A. hydrophila* (10^6^ CFU/ml) suspended in PBS were incubated with topmouth culter LEAP-2 (1 × MIC), ampicillin (1 mg/ml) or PBS (vehicle) in the presence of SYTOX (0.1 μM) at 37°C for 15 min on a shaking table. Fluorescence intensity was monitored and presented as relative fluorescence unit (RFU).

To further confirm the membrane-disruption mechanism of topmouth culter LEAP-2, *A. hydrophila* were incubated with LEAP-2 (1 × MIC, 18.75 μg/ml, equal to 4.0 μM) in the presence of SYTOX Green and the fluorescence intensity was monitored. Compared to PBS-treated bacteria, the fluorescence intensity dramatically increased in 16 min after the addition of topmouth culter LEAP-2, while no significant increment of fluorescence intensity was observed in ampicillin-treated (200 μg/ml, equal to 572.4 μM) bacteria (Figure 5). The results indicated that topmouth culter LEAP-2 treatment increased the uptake of fluorescent dye by *A. hydrophila*, and the bacterial nucleic acids were subsequently stained with SYTOX Green, resulting in an increment of fluorescence intensity. Combined with results observed by SEM assay, LEAP-2 was demonstrated to evidently cause pore formation on the bacterial membrane.

### Topmouth Culter LEAP-2 Showed None Inducible Drug Resistance to Bacteria

Antimicrobial peptides are less likely to induce bacterial drug resistance and were usually selected as promising candidates for peptide antibiotic development (Mwangi et al., 2019). We herein detected whether topmouth culter LEAP-2 induce drug resistance against aquatic pathogenic bacteria. An ampicillin sensitive strain, *V. parahaemolyticus*, was exposed to sub-therapeutic doses of topmouth culter LEAP-2 or ampicillin (control) over 10 passages to simulate drug resistance (Yang et al., 2019). As shown in Table 4, PBS (vehicle) did not affect the drug resistance of *V. parahaemolyticus* against topmouth culter LEAP-2 or ampicillin, and resistance of *V. parahaemolyticus* against topmouth culter LEAP-2 did not readily develop with sub-therapeutic treatment of the peptide, as shown by the consistent MIC values obtained over 10 passages. In contrast, the MIC value of ampicillin against *V. parahaemolyticus* increased from 18.75 to 300 μg/ml (increased by 16-fold), which indicated that resistance of *V. parahaemolyticus* against ampicillin did develop with sub-therapeutic treatment.

**Table 4:** Induced drug resistance test of topmouth culter LEAP-2 and ampicillin against *Vibrio parahaemolyticus*.

Stimulation drug	Tested drug	MIC(passage 0) (μg/ml)*^a^*	MIC(passage 10) (μg/ml)*^a^*
PBS (vehicle)	LEAP-2	75	75
LEAP-2	LEAP-2	75	75
PBS (vehicle)	Ampicillin	18.75	18.75
Ampicillin	Ampicillin	18.75	300

Drug resistance was induced in *Vibrio parahaemolyticus* by repeated treatment with ampicillin, topmouth culter LEAP-2 or PBS for 10 passages and tested via MIC measurement. For each passage, *V. parahaemolyticus* was exposed to a sub-MIC concentration of ampicillin or topmouth culter LEAP-2 (1/8 MIC at that particular passage) until growing to the log phase, and the MIC values of topmouth culter LEAP-2 and ampicillin against *V. parahaemolyticus* were determined.

a These concentrations represent mean values from three independent experiments.

### Topmouth Culter LEAP-2 Delayed Ampicillin-Induced Bacterial Drug Resistance

The combinational usage of AMPs and traditional antibiotics might be a promising way to overcome the emergence of bacterial drug-resistance and generate a greater antimicrobial effect (Yang et al., 2019). Therefore, we detected ampicillin-induced drug resistance against *V. parahaemolyticus* as mentioned above in the presence or absence of a constant concentration of topmouth culter LEAP-2 (18.75 μg/ml, 1/4 MIC of LEAP-2 against *V. parahaemolyticus*). As shown in Table 5, treatment of *V. parahaemolyticus* with sub-therapeutic dose of ampicillin readily induced ampicillin resistance with the MIC value increasing from 18.75 to 300 μg/ml (increased by 16-fold), while treatment of *V. parahaemolyticus* with sub-therapeutic concentration of ampicillin and a constant concentration of LEAP-2 (18.75 μg/ml, 1/4 MIC of LEAP-2 against *V. parahaemolyticus*) just induced a modest ampicillin resistance with the MIC value increasing from 18.75 to 37.5 μg/ml (increased by 2-fold), which indicated that topmouth culter LEAP-2 delayed ampicillin-induced drug resistance against *V. parahaemolyticus*.

**Table 5:** Topmouth culter LEAP-2 delayed the ampicillin-induced drug resistance against *V. parahaemolyticus*.

Stimulation drug	Tested drug	MIC(passage 0) (μg/ml)*^a^*	MIC(passage 10) (μg/ml)*^a^*
Ampicillin	Ampicillin	18.75	300
Ampicillin + LEAP-2	Ampicillin	18.75	37.5

*V. parahaemolyticus* were passaged for 10 times in the presence of ampicillin and topmouth culter LEAP-2. For each passage, *V. parahaemolyticus* was exposed to a sub-MIC concentration of ampicillin (1/8 MIC at that particular passage) and a constant concentration of topmouth culter LEAP-2 (18.75 μg/ml, 1/4 MIC of LEAP-2 against *V. parahaemolyticus*) until growing to the log phase, and the MIC values of ampicillin against *V. parahaemolyticus* were determined via MIC assay.

a These concentrations represent mean values from three independent experiments.

### Topmouth Culter LEAP-2 Sensitized Drug-Resistant Bacteria to Antibiotic

To evaluate the potential of combinational usage of topmouth culter LEAP-2 with traditional antibiotics, we detected the MIC values of ampicillin against eight aquatic bacterial strains in the presence of a constant concentration of topmouth culter LEAP-2 (1/4 MIC of LEAP-2 against respective bacterial strain) or a same volume of PBS (vehicle), including ampicillin-sensitive and ampicillin-resistant strains. As shown in Table 6, the MIC values of ampicillin-sensitive strains (*V. harveyi* and *V. parahaemolyticus*) were decreased by 2–4 folds in the presence of topmouth culter LEAP-2. In addition, ampicillin-resistant strains (MIC value >200 μg/ml), including *A. sobria*, *A. hydrophila*, *V. anguillarum*, *V. vulnificus*, *V. splendidus*, and *V. cholerae*, were sensitive to ampicillin in the presence of topmouth culter LEAP-2 with MIC value <200 μg/ml. These results indicated that topmouth culter LEAP-2 could sensitize aquatic pathogenic bacteria (including drug-resistant aquatic pathogenic bacteria) to antibiotic.

**Table 6:** MIC assay of ampicillin against fish pathogenic bacteria in the presence of topmouth culter LEAP-2.

Microorganisms	MIC (μg/ml)*^a^*	
	Ampicillin + PBS (vehicle)*^b^*	Ampicillin + LEAP-2*^c^*
*Aeromonas sobria*	>200	150
*Aeromonas hydrophila*	>200	75
*Vibrio harveyi*	37.5	9.38
*Vibrio parahaemolyticus*	18.75	9.38
*Vibrio anguillarum*	>200	>200
*Vibrio vulnificus*	>200	150
*Vibrio splendidus*	>200	75
*Vibrio cholerae*	>200	75

MIC values of ampicillin against each bacterial strain was tested in the presence of topmouth culter LEAP-2.

a MIC, minimal inhibitory concentration. These concentrations represent mean values from three independent experiments.

b An equal volume of PBS (vehicle) served as negative control.

c The concentration of the peptide used for each bacterial strain is 1/4 MIC of topmouth culter LEAP-2 against respective bacterial strain.

### Topmouth Culter LEAP-2 Showed Synergistic Effects With Ampicillin

As mentioned above, topmouth culter LEAP-2 could delay ampicillin-induced drug resistance and sensitize drug-resistant aquatic pathogenic bacteria to ampicillin, indicating that topmouth culter LEAP-2 might have an additive effect or synergistic effect with ampicillin. To investigate which effect was involved in the interaction of topmouth culter LEAP-2 with ampicillin, a chequerboard assay was performed as shown in Table 7. The combinational usage of topmouth culter LEAP-2 and ampicillin generated an FICI value of 0.375 against *V. harveyi*, and an FICI value of 0.5 against *V. parahaemolyticus*, indicating that topmouth culter LEAP-2 exhibited synergistic effects with ampicillin. Of note, peptide-ampicillin used in combination produced a lower minimum effective concentration for each agent. Combined with the membrane-disrupted action of topmouth culter LEAP-2 mentioned above, the synergistic effects between LEAP-2 and ampicillin is likely attributed to the LEAP-2-mediated permeation of ampicillin from outer membrane to cytoplasmic targets.

**Table 7:** Chequerboard assay of ampicillin and topmouth culter LEAP-2 against fish pathogenic bacteria.

Microorganism	Drug combination	MIC (μg/ml)		FIC	FICI*^a^*
		Alone	Combination		
*Vibrio harveyi*	Ampicillin	37.5	4.69	0.125	0.375
	LEAP-2	75	18.75	0.25	
*Vibrio parahaemolyticus*	Ampicillin	18.75	4.69	0.25	0.5
	LEAP-2	75	18.75	0.25	

a The fractional inhibitory concentration index (FICI) was calculated for each combination using this equation: FICI = FIC_A_ + FIC_B_, where FIC_A_ = MIC of drug A in combination/MIC of drug A alone, and FIC_B_ = MIC of drug B in combination/MIC of drug B alone. FICI of ≤0.5 was interpreted as synergy, 0.5 < FICI ≤ 1.0 as additive, 1.0 < FICI ≤4.0 as indifferent, and FICI > 4.0 as antagonism. These concentrations represent mean values of three independent experiments performed in duplicates.

### Topmouth Culter LEAP-2 Alleviated Ampicillin-Resistant Bacterial Infection and Enhanced the Therapeutic Efficacy of Ampicillin Against Antibiotic-Resistant Bacteria *in vivo*

**Figure 6:**
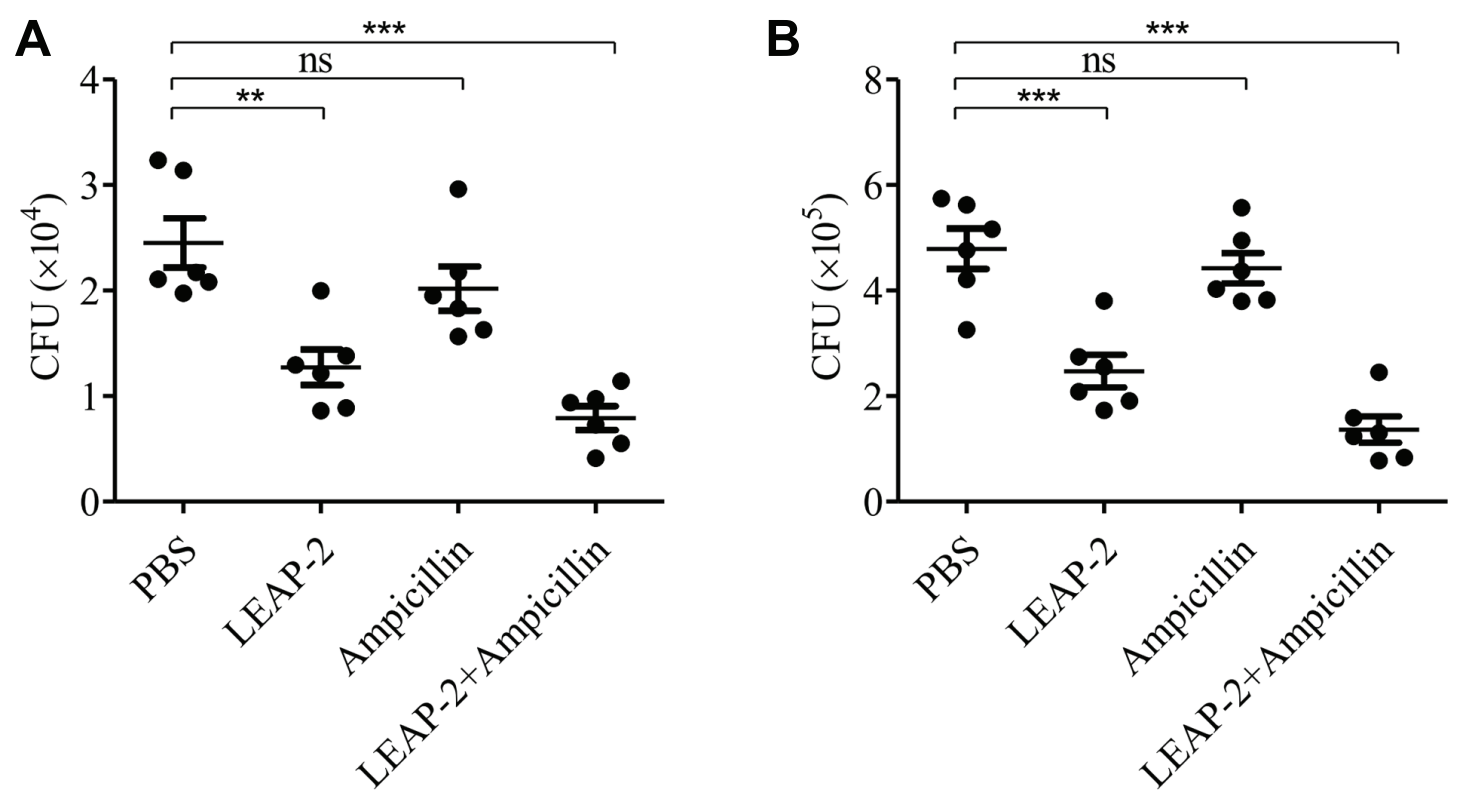
Antimicrobial activity and combinational usage of topmouth culter LEAP-2 with ampicillin *in vivo*. **(A)** Topmouth culter (350–450 g, *n* = 6) were intraperitoneally challenged with *A. hydrophila* (100 μl, 2 × 10^7^ CFU/ml), and immediately intraperitoneally administrated with LEAP-2 (10 mg/kg), ampicillin (10 mg/kg), LEAP-2 and ampicillin used in combination (10 mg/kg each), or an equal volume of PBS (vehicle), respectively. At 8 h post infection, bacterial loads in the peritoneal lavage of topmouth culter were counted. **(B)** C57BL/6 mice (female, 18–20 g, *n* = 6) were intraperitoneally challenged with *A. hydrophila* (2 × 10^7^ CFUs/mouse). Topmouth culter LEAP-2 (10 mg/kg) or an equal volume of PBS (vehicle) was intraperitoneally administered into mice post bacterial infection. At 18 h post infection, bacterial loads in the peritoneal lavage of mice were counted. ns, not significant, ^**^ *p* < 0.01, ^***^ *p* < 0.001.

The *in vitro* experiments indicated that topmouth culter LEAP-2 had an excellent combinational usage potential in treating drug-resistant bacterial infection. We next evaluated its anti-infective effects against *A. hydrophila* (resistant to ampicillin) combined with ampicillin *in vivo*. Compared with PBS-treated topmouth culter, the intraperitoneal bacterial loads of topmouth culter were significantly reduced after the administration of LEAP-2 (10 mg/kg), and the CFUs in the peritoneal lavage of topmouth culter were reduced by 47.8% (Figure 6A). While ampicillin (10 mg/kg) did not significantly reduce the intraperitoneal bacterial loads of topmouth culter (Figure 6A). As expected, the combinational usage of topmouth culter LEAP-2 (10 mg/kg) with ampicillin (10 mg/kg) showed the best efficacy against ampicillin-resistant *A. hydrophila* infection, and the CFUs in the peritoneal lavage of topmouth culter were reduced by 67.2% (Figure 6A). A similar result was observed in mice. Topmouth culter LEAP-2 (10 mg/kg) significantly reduced the loads of ampicillin-resistant *A. hydrophila* in mouse peritoneal lavage, reducing about 46.9% CFUs (Figure 6B). But ampicillin (10 mg/kg) did not markedly affect the bacterial loads in mice (Figure 6B). Whereas the combinational injection of topmouth culter LEAP-2 (10 mg/kg) and ampicillin (10 mg/kg) exhibited the best therapeutic efficacy among these groups, reducing about 72.1% CFUs (Figure 6B). These results suggested that topmouth culter LEAP-2 markedly alleviated ampicillin-resistant bacterial infection *in vivo* and enhanced the therapeutic efficacy of antibiotic against antibiotic-resistant bacterial infection *in vivo*.

## Discussion

In recent years, the research activities about AMPs have gained many achievements throughout the world. Due to the small MW and simple molecular structure of these peptides, it is easy to produce these peptides with low production cost. Therefore, more and more researchers are trying their best to develop peptide drugs derived from AMPs. In mammals, AMPs showed diverse biologic activities such as enodotoxin neutralization, anti-inflammation, chemotaxis of leukocytes, promotion of wound healing, and induction of angiogenesis (Hancock et al., 2016). They have been regarded as very attractive therapeutic molecules. Especially, AMPs have been considered as most promising agents for production of new generation antibiotics. Many peptide antibiotics derived from AMPs are undergoing clinical trials. For example, four cathelicidin analogs, omiganan (bovine indolicidin analogue, MBI-226, CPI226), MX-594AN (bovine indolicidin analogue), and iseganan (porcine protegrin-1 analogue and IB-367) are undergoing clinical trials as novel anti-infective agents (Chen et al., 2019).

In case of fishes, AMPs are crucial members of innate immunity that provide first line of host defense. AMPs from fishes was initially characterized from the secretion of Moses sole fish *Pardachirus marmoratus* in 1980, which is called pardaxin (Primor and Tu, 1980). Pardaxin was first described as a toxic peptide and was used as a shark repellent (Primor and Tu, 1980). Later, pardaxin was demonstrated to possess a potent pore-formation property (Lazarovici et al., 1986) and a high antibacterial activity (Oren and Shai, 1996). Over the past decades, several AMP families, like piscidins, cathelicidins, defensins, and hepcidins (also known as liver expressed APs, LEAPs), have been identified from fishes (Shabir et al., 2018). In general, these AMPs from fishes showed direct antimicrobial potency against bacteria, fungi, viruses and parasites. Almost all AMPs from fishes showed direct antibacterial or bacteriostatic activities against several Gram-negative and Gram-positive bacterial strains (Rajanbabu and Chen, 2011). Some AMPs from fishes displayed anti-fungal activities. Piscidin 2 secreted by striped bass could act as a fungicide by disrupting fungal membranes (Sung et al., 2008). A few AMPs from fishes exhibited antiviral activities. Rainbow trout *β*-defensin 1 showed antiviral activity against viral hemorrhagic septicemia virus (VHSV) infection (Falco et al., 2008). AMPs from fishes also showed anti-parasitic functions. Pisicidin 2 isolated from hybrid striped bass showed potent anti-parasitic effects against three protistan ectoparasites of marine fish and one ciliate ectoparasite of freshwater fish (Colorni et al., 2008). In addition, AMPs from fishes also acted as immunostimulants that modulated the anti-bacterial immune response and basal immune response of fish. Cathelicidins from *Brachymystax lenok* and *Gadus morhua* exhibited potent immunoregulatory activities by inhibition of pro-inflammatory cytokine (tumor necrosis factor-α, interleukin-1β, and interleukin-6) expression in bacteria-infected zebrafish and induction of chemokine (interleukin-8) expression in healthy zebrafish. In this study, topmouth culter LEAP-2 exhibited direct anti-bacterial activities against aquatic pathogenic bacteria *via* a membrane-disruption mechanism. The direct anti-bacterial properties of topmouth culter LEAP-2 are consistent with those observed from other fish-derived AMPs. In addition, it also showed potent anti-bacterial activities in different temperatures (Supplementary Table S1) and different buffers (Supplementary Table S2) that mimicked the fish environment. In the present study, we did not test the anti-fungal, anti-viral, anti-parasitic, and immunomodulatory functions of topmouth culter LEAP-2, which need to be further elucidated in future.

Because of the widespread of antibiotics, the increasing antibiotic-resistant bacterial strains have become a severe threat to fish farming. In the past decades, many researchers tried to develop the substitutes of antibiotics in aquaculture industry aquaculture, which will control the diseases in cultured aquatic animals induced by the spread of antibiotic-resistant bacterial strains. AMPs are largely membrane-active agents that alter bacterial membrane integrity by inducing pore formation (Khara et al., 2014). The membrane-disruption mechanism of AMPs against bacteria are different from the mechanism of antibiotics against bacteria. Compared to the mechanism of action of antibiotics, the membrane-disrupted mechanism of AMPs against bacteria is non-specific, which makes host AMPs have broad anti-bacterial spectrum, including antibiotic-sensitive and antibiotic-resistant bacterial strains. Besides, this non-specific anti-bacterial mechanism is difficult to induce the bacterial resistance against AMPs. In our study, topmouth culter LEAP-2 significantly disrupted membrane integrity of aquatic pathogenic bacterial membrane and showed none inducible drug-resistance to aquatic pathogenic bacteria. Interestingly, topmouth culter LEAP-2 delayed ampicillin-induced drug resistance against *V. parahaemolyticus*, sensitized ampicillin-resistant aquatic pathogenic bacteria to ampicillin, and generated synergistic effects with ampicillin. It is more likely that the induction of pore formation on bacterial membrane mediates the permeation of ampicillin from outer membrane to cytoplasmic targets. *In vivo* test proved that topmouth culter LEAP-2 did inhibit ampicillin-resistant *A. hydrophila* infection and did enhance the therapeutic efficacy of ampicillin against antibiotic-resistant *A. hydrophila* infection. Since the membrane-disrupted mechanism is a non-specific way, topmouth culter LEAP-2-induced pore formation on bacterial membrane possibly also promotes the permeation of other antibiotics from bacterial outer membrane to cytoplasmic targets, thereby generating interactive effects with other antibiotics. These findings made topmouth culter LEAP-2 an excellent candidate for the combinational usage with antibiotic, and the combinational usage of topmouth culter LEAP-2 and antibiotic may slow the progression of antibiotic-resistant aquatic pathogenic bacteria and even prevent the emergence of antibiotic-resistant aquatic pathogenic bacteria.

In conclusion, we identified a novel AMP from topmouth culter that belonged to LEAP-2 family. Topmouth culter LEAP-2 showed antimicrobial activities against aquatic pathogenic bacteria with rapid bactericidal speed. It dramatically induced bacterial membrane permeabilization by eliciting pore formation on bacterial membrane. It showed none inducible drug resistance to aquatic pathogenic bacteria and efficiently delayed ampicillin-induced drug resistance against aquatic pathogenic bacteria. It could sensitize ampicillin-resistant aquatic pathogenic bacterial strains to ampicillin and could generate synergistic effect with ampicillin. *In vivo*, topmouth culter LEAP-2 markedly alleviated ampicillin-resistant bacterial infection and enhanced the therapeutic efficacy of antibiotic against antibiotic-resistant bacterial infection. Our findings highlighted its development potential for antibiotic-resistant bacterial infection in aquaculture industry.

## Data Availability Statement

The datasets presented in this study can be found in online repositories. The names of the repository/repositories and accession number(s) can be found in the article/Supplementary Material.

## Ethics Statement

The animal study was reviewed and approved by Animal Care and Use Committee and the Ethical Committee of Soochow University (SYXK2017-0043). Written informed consent was obtained from the owners for the participation of their animals in this study.

## Author Contributions

YC, JW, and HC contributed to experimental studies and data analysis. LW designed the experiment. FX, WX, and LW wrote and revised the manuscript and contributed to financial support. YD, YW, and HY contributed to the discussion. All authors contributed to the article and approved the submitted version.

### Conflict of Interest

The authors declare that the research was conducted in the absence of any commercial or financial relationships that could be construed as a potential conflict of interest.

## Footnotes

^1^http://web.expasy.org/translate/

^2^http://www.ncbi.nlm.nih.gov/

^3^http://embnet.vital-it.ch/software/ClustalW.html

^4^http://web.expasy.org/compute_pi/

## References

[ref1] Bao B. Peatman E. Xu P. Li P. Zeng H. He C.. (2006). The catfish liver-expressed antimicrobial peptide 2 (LEAP-2) gene is expressed in a wide range of tissues and developmentally regulated. Mol. Immunol. 43, 367–377. 10.1016/j.molimm.2005.02.014, PMID: 16310050

[ref2] Chen J. Chen Q. Lu X. J. Chen J. (2016). The protection effect of LEAP-2 on the mudskipper (*Boleophthalmus pectinirostris*) against Edwardsiella tarda infection is associated with its immunomodulatory activity on monocytes/macrophages. Fish Shellfish Immunol. 59, 66–76. 10.1016/j.fsi.2016.10.028, PMID: 27765699

[ref3] Chen J. Lv Y. P. Dai Q. M. Hu Z. H. Liu Z. M. Li J. H. (2019). Host defense peptide LEAP-2 contributes to monocyte/macrophage polarization in barbel steed (*Hemibarbus labeo*). Fish Shellfish Immunol. 87, 184–192. 10.1016/j.fsi.2019.01.015, PMID: 30641185

[ref4] Chen C. Wang A. Zhang F. Zhang M. Yang H. Li J.. (2019). The protective effect of fish-derived cathelicidins on bacterial infections in zebrafish, *Danio rerio*. Fish Shellfish Immunol. 92, 519–527. 10.1016/j.fsi.2019.06.029, PMID: 31202967

[ref5] Colorni A. Ullal A. Heinisch G. Noga E. J. (2008). Activity of the antimicrobial polypeptide piscidin 2 against fish ectoparasites. J. Fish Dis. 31, 423–432. 10.1111/j.1365-2761.2008.00922.x, PMID: 18471098

[ref6] Dong H. Chen W. Sun C. Sun J. Wang Y. Xie C.. (2017). Identification, characterization of selenoprotein W and its mRNA expression patterns in response to somatostatin 14, cysteamine hydrochloride, 17beta-estradiol and a binary mixture of 17beta-estradiol and cysteamine hydrochloride in topmouth culter (Erythroculter ilishaeformis). Fish Physiol. Biochem. 43, 115–126. 10.1007/s10695-016-0272-9, PMID: 27506211

[ref7] Dong H. Wei Y. Xie C. Zhu X. Sun C. Fu Q.. (2018). Structural and functional analysis of two novel somatostatin receptors identified from topmouth culter (Erythroculter ilishaeformis). Comp. Biochem. Physiol. C Toxicol. Pharmacol. 210, 18–29. 10.1016/j.cbpc.2018.04.004, PMID: 29698686

[ref8] Falco A. Chico V. Marroqui L. Perez L. Coll J. M. Estepa A. (2008). Expression and antiviral activity of a beta-defensin-like peptide identified in the rainbow trout (*Oncorhynchus mykiss*) EST sequences. Mol. Immunol. 45, 757–765. 10.1016/j.molimm.2007.06.358, PMID: 17692376

[ref9] Guo Z. Qiao X. Cheng R. Shi N. Wang A. Feng T.. (2017). As-CATH4 and 5, two vertebrate-derived natural host defense peptides, enhance the immuno-resistance efficiency against bacterial infections in Chinese mitten crab, *Eriocheir sinensis*. Fish Shellfish Immunol. 71, 202–209. 10.1016/j.fsi.2017.10.015, PMID: 29017942

[ref10] Hancock R. E. Haney E. F. Gill E. E. (2016). The immunology of host defence peptides: beyond antimicrobial activity. Nat. Rev. Immunol. 16, 321–334. 10.1038/nri.2016.29, PMID: 27087664

[ref11] Jin L. Bai X. Luan N. Yao H. Zhang Z. Liu W.. (2016). A designed tryptophan- and lysine/arginine-rich antimicrobial peptide with therapeutic potential for clinical antibiotic-resistant *Candida albicans* vaginitis. J. Med. Chem. 59, 1791–1799. 10.1021/acs.jmedchem.5b01264, PMID: 26881456

[ref12] Khara J. S. Wang Y. Ke X. Y. Liu S. Newton S. M. Langford P. R.. (2014). Anti-mycobacterial activities of synthetic cationic alpha-helical peptides and their synergism with rifampicin. Biomaterials 35, 2032–2038. 10.1016/j.biomaterials.2013.11.035, PMID: 24314557

[ref13] Lazarovici P. Primor N. Loew L. M. (1986). Purification and pore-forming activity of two hydrophobic polypeptides from the secretion of the Red Sea Moses sole (*Pardachirus marmoratus*). J. Biol. Chem. 261, 16704–16713., PMID: 3782138

[ref14] Li H. X. Lu X. J. Li C. H. Chen J. (2015). Molecular characterization of the liver-expressed antimicrobial peptide 2 (LEAP-2) in a teleost fish, *Plecoglossus altivelis*: antimicrobial activity and molecular mechanism. Mol. Immunol. 65, 406–415. 10.1016/j.molimm.2015.02.022, PMID: 25749706

[ref15] Liang T. Ji W. Zhang G. R. Wei K. J. Feng K. Wang W. M.. (2013). Molecular cloning and expression analysis of liver-expressed antimicrobial peptide 1 (LEAP-1) and LEAP-2 genes in the blunt snout bream (*Megalobrama amblycephala*). Fish Shellfish Immunol. 35, 553–563. 10.1016/j.fsi.2013.05.021, PMID: 23748217

[ref16] Liu T. Gao Y. Wang R. Xu T. (2014). Characterization, evolution and functional analysis of the liver-expressed antimicrobial peptide 2 (LEAP-2) gene in miiuy croaker. Fish Shellfish Immunol. 41, 191–199. 10.1016/j.fsi.2014.08.010, PMID: 25180825

[ref17] Liu F. Li J. L. Yue G. H. Fu J. J. Zhou Z. F. (2010). Molecular cloning and expression analysis of the liver-expressed antimicrobial peptide 2 (LEAP-2) gene in grass carp. Vet. Immunol. Immunopathol. 133, 133–143. 10.1016/j.vetimm.2009.07.014, PMID: 19716607

[ref18] Mwangi J. Yin Y. Wang G. Yang M. Li Y. Zhang Z.. (2019). The antimicrobial peptide ZY4 combats multidrug-resistant Pseudomonas aeruginosa and Acinetobacter baumannii infection. Proc. Natl. Acad. Sci. U. S. A. 116, 26516–26522. 10.1073/pnas.1909585117, PMID: 31843919 PMC6936460

[ref19] Oren Z. Shai Y. (1996). A class of highly potent antibacterial peptides derived from pardaxin, a pore-forming peptide isolated from Moses sole fish *Pardachirus marmoratus*. Eur. J. Biochem. 237, 303–310. 10.1111/j.1432-1033.1996.0303n.x, PMID: 8620888

[ref20] Primor N. Tu A. T. (1980). Conformation of pardaxin, the toxin of the flatfish *Pardachirus marmoratus*. Biochim. Biophys. Acta 626, 299–306. 10.1016/0005-2795(80)90124-5, PMID: 7213649

[ref21] Rajanbabu V. Chen J. Y. (2011). Applications of antimicrobial peptides from fish and perspectives for the future. Peptides 32, 415–420. 10.1016/j.peptides.2010.11.005, PMID: 21093512

[ref22] Shabir U. Ali S. Magray A. R. Ganai B. A. Firdous P. Hassan T.. (2018). Fish antimicrobial peptides (AMP’s) as essential and promising molecular therapeutic agents: a review. Microb. Pathog. 114, 50–56. 10.1016/j.micpath.2017.11.039, PMID: 29180291

[ref23] Sung W. S. Lee J. Lee D. G. (2008). Fungicidal effect and the mode of action of piscidin 2 derived from hybrid striped bass. Biochem. Biophys. Res. Commun. 371, 551–555. 10.1016/j.bbrc.2008.04.107, PMID: 18445475

[ref24] Wei L. Che H. Han Y. Lv J. Mu L. Lv L.. (2015). The first anionic defensin from amphibians. Amino Acids 47, 1301–1308. 10.1007/s00726-015-1963-8, PMID: 25792112

[ref25] Wei L. Yang J. He X. Mo G. Hong J. Yan X.. (2013). Structure and function of a potent lipopolysaccharide-binding antimicrobial and anti-inflammatory peptide. J. Med. Chem. 56, 3546–3556. 10.1021/jm4004158, PMID: 23594231

[ref26] Wei L. Yang Y. Zhou Y. Li M. Yang H. Mu L.. (2018). Anti-inflammatory activities of *Aedes aegypti* cecropins and their protection against murine endotoxin shock. Parasit. Vectors 11:470. 10.1186/s13071-018-3000-8, PMID: 30107813 PMC6092832

[ref27] Xiong J. B. Nie L. Chen J. (2019). Current understanding on the roles of gut microbiota in fish disease and immunity. Zool. Res. 40, 70–76. 10.24272/j.issn.2095-8137.2018.069, PMID: 29976843 PMC6378566

[ref28] Yang G. Guo H. Li H. Shan S. Zhang X. Rombout J. H.. (2014). Molecular characterization of LEAP-2 cDNA in common carp (*Cyprinus carpio* L.) and the differential expression upon a Vibrio anguillarum stimulus; indications for a significant immune role in skin. Fish Shellfish Immunol. 37, 22–29. 10.1016/j.fsi.2014.01.004, PMID: 24418455

[ref29] Yang Y. Liu Z. He X. Yang J. Wu J. Yang H.. (2019). A small mycobacteriophage-derived peptide and its improved isomer restrict mycobacterial infection via dual mycobactericidal-immunoregulatory activities. J. Biol. Chem. 294, 7615–7631. 10.1074/jbc.RA118.006968, PMID: 30894414 PMC6514635

[ref30] Yousefi S. Hoseinifar S. H. Paknejad H. Hajimoradloo A. (2018). The effects of dietary supplement of galactooligosaccharide on innate immunity, immune related genes expression and growth performance in zebrafish (*Danio rerio*). Fish Shellfish Immunol. 73, 192–196. 10.1016/j.fsi.2017.12.022, PMID: 29258754

[ref31] Zasloff M. (2002). Antimicrobial peptides of multicellular organisms. Nature 415, 389–395. 10.1038/415389a, PMID: 11807545

[ref32] Zasloff M. (2019). Antimicrobial peptides of multicellular organisms: my perspective. Adv. Exp. Med. Biol. 1117, 3–6. 10.1007/978-981-13-3588-4_1, PMID: 30980349

[ref33] Zhang Y. A. Zou J. Chang C. I. Secombes C. J. (2004). Discovery and characterization of two types of liver-expressed antimicrobial peptide 2 (LEAP-2) genes in rainbow trout. Vet. Immunol. Immunopathol. 101, 259–269. 10.1016/j.vetimm.2004.05.005, PMID: 15350756

[ref34] Zhu L. Y. Nie L. Zhu G. Xiang L. X. Shao J. Z. (2013). Advances in research of fish immune-relevant genes: a comparative overview of innate and adaptive immunity in teleosts. Dev. Comp. Immunol. 39, 39–62. 10.1016/j.dci.2012.04.001, PMID: 22504163

