# Supplementary material for: Anti-infective Effects of a Fish-Derived Antimicrobial Peptide Against Drug-Resistant Bacteria and Its Synergistic Effects With Antibiotic
Source: Front Microbiol. 2020 Nov 23;11:602412. doi: 10.3389/fmicb.2020.602412 (PMC7719739; doi:10.3389/fmicb.2020.602412)
Supplement: Supplementary file 1 [file Data_Sheet_1.docx]

**Supplemental tables**

**Table S1. Antimicrobial activities of topmouth culter LEAP-2 at different temperatures**

| Microorganisms | MIC (μg/ml)*^a^* | |
| --- | --- | --- |
|  | 25℃ | 37℃ |
| *Aeromonas sobria* | 75 | 75 |
| *Aeromonas hydrophila* | 18.75 | 18.75 |
| *Vibrio harveyi* | 75 | 75 |
| *Vibrio parahaemolyticus* | 75 | 75 |

*^a^*MIC: minimal inhibitory concentration. These concentrations represent mean values from three independent experiments.

**Table S2. Antimicrobial activities of topmouth culter LEAP-2 dissolved in different buffers**

| Microorganisms | MIC (μg/ml)*^a^* | | |
| --- | --- | --- | --- |
|  | PBS | H_2_O | NaCl (0.7%) |
| *Aeromonas sobria* | 75 | 75 | 75 |
| *Aeromonas hydrophila* | 18.75 | 18.75 | 18.75 |
| *Vibrio harveyi* | 75 | 75 | 75 |
| *Vibrio parahaemolyticus* | 75 | 75 | 75 |

*^a^*MIC: minimal inhibitory concentration. These concentrations represent mean values from three independent experiments.
